# 

**Published:** 1857-02

**Authors:** 


ARTICLE I.—Report of a Case of Monstrosity, with a Surgical operation. By E. C. MOYER, M. D., Talbotton, Ga.,.......... 321

ARTICLE II.—Report of a Complicated Case of Dislocation of the Knee Joint, with Remarks. By V.H. TALIAFERRO, M. D., Atlanta, Ga.,...... 323

ARTICLE III.—Homœopathy. By ROBERT E. CAMPBELL, M. D., Benton, Lowndes County, Alabama,.......... 329

ARTICLE IV.—Report from the Case-Book of THOS. S. POWELL, M. D. Sparta, Georgia,.......... 333

ARTICLE V.—What is the matter? Or, Report of a Case by Dr. JOHN COSTON, White House, Henry County, Georgia,.......... 336

ARTICLE VI.—The Syphilisation of Children. • By B. W. BOECK, Professor de Medicine, a l'Universite de Christiana. Translated for the Atlanta Medical and Surgical Journal, by J. J. WEST, M. D., Demonstrator of Anatomy, Savannah Medical College,.......... 338

### SELECTIONS.

On the Evil Effects of Marriages of Consanguinity,.......... 351

Biographical Sketch of SAMUEL HENRY DICKSON, M. D., LL. D.,.......... 360

### EDITORIAL AND MISCELLANEOUS.

Editorial Correspondence,.......... 363

Case of Hon. Charles Sumner,.......... 372

The College Edifice, &c.,.......... 374

The present number of the Atlanta Medical Journal,.......... 376

The accessibility of the Journal to Correspondents,.......... 376

Back numbers of the present volume,.......... 376

Physiology,.......... 376

Tragic End of John Hunter,.......... 382

Prospect of the additional interest of our Journal,.......... 383

Receipts,.......... 383

Prospectus of North Carolina Journal of Medicine and Surgery,.......... 384

Meteorological Observations for January, 1857, Atlanta, Ga.,.......... 384

